# Validation of miRNA-mRNA interactions by electrophoretic mobility shift assays

**DOI:** 10.1186/1756-0500-6-454

**Published:** 2013-11-12

**Authors:** Anna Solé, Núria Mencia, Xenia Villalobos, Véronique Noé, Carlos J Ciudad

**Affiliations:** 1 Department of Biochemistry and Molecular Biology, School of Pharmacy, University of Barcelona, Avenue Diagonal 643, Barcelona E-08028, Spain

**Keywords:** 3′-UTR, Binding assay, miRNA, Target validation, EMSA

## Abstract

**Background:**

MicroRNAs are small non-coding RNAs involved in gene expression regulation by targeting specific regions in the 3′-UTR of the mRNA of their target genes. This binding leads to a decrease in the protein levels of such genes either by mRNA degradation or mRNA destabilization and translation inhibition. The interaction between a miRNA and its target mRNAs is usually studied by co-transfection of a reporter expression vector containing the 3′-UTR region of the mRNA and an inhibitory or precursor molecule for the miRNA. This approach, however, does not measure the direct and physical interaction between a miRNA and a specific mRNA.

**Findings:**

RNA molecules corresponding to miR-224 and to the 3′-UTR of SLC4A4 were incubated together and their interaction studied under different binding conditions using electrophoretic mobility shift assays. A direct and specific interaction between miR-224 and SLC4A4 mRNA was observed. This interaction was abolished in the presence of competitors.

**Conclusions:**

In this study, we explored a new application for the electrophoretic mobility shift assay and we demonstrated that it is a useful alternative method to assess, in a direct and specific manner, whether a miRNA binds to a specific predicted target mRNA.

## Introduction

MiRNAs are a new class of small, non protein-encoding RNAs [1,2] responsible for gene expression regulation at a post-transcriptional level. MiRNAs recognize and bind to the 3′-UTR of the mRNA of their target genes. Only 6-7nt of the miRNA (miRNA seed) are complementary to the target mRNA. Imperfect base pairing between miRNAs and their target mRNAs leads to repression of translation and/or deadenylation, followed by destabilization of the target [3]. Many cellular pathways are regulated by miRNAs such as development, proliferation, differentiation, cell fate determination, apoptosis, signal transduction, organ development, host-viral interactions and tumorigenesis [4]. MiRNAs play key roles in the pathogenesis of cancer and many miRNAs have been shown to function as either oncomirs or tumor suppressors [5-11] (for review see [12]). Furthermore, miRNAs have an important role in the development of chemosensitivity or chemoresistance in different types of cancer (summarized in [13]). The prediction of target genes for a given miRNA is carried out using bioinformatic algorithms that require experimental validation. The interaction of the miRNA target gene with a specific miRNA is usually studied by co-transfection of an inhibitory molecule (anti-miR) or miRNA precursor molecule together with a reporter vector containing the 3′-UTR region of the gene. Although this method suggests a physical and functional interaction, it does not prove a direct interaction of the target gene with the miRNA [14]. More complex methods based on immunoprecipitation of the complex miRNA/mRNA have been used to address this issue [15,16].

In a previous study, we studied the role of miR-224 in methotrexate resistance in HT-29 colon cancer cells. The underexpression of miR-224 resulted in a decrease in MTX sensitivity and an increase in the mRNA levels of three genes (*CDS2*, *HSPC159* and *SLC4A4*) whose role in MTX resistance was also functionally validated. After confirming within the cell that miR-224 was functionally regulating these three genes, we validated the direct and physical interaction between miR-224 and *CDS2*, *HSPC159* or *SLC4A4* using binding assays [17].

EMSA or electrophoretic mobility shift assays are widely used to confirm the binding of a nucleic acid to a protein or another nucleic acid [18-20]. However, there have been no reports regarding the use of EMSA to study the interaction of a miRNA with its target. Therefore, we propose EMSA as an alternative method to validate in a direct and specific fashion whether or not a miRNA binds to its target genes. This study describes the experiments carried out to develop this methodology.

## Materials and methods

### Materials

For binding assays, the following molecules were used: *miR-224*, RNA sequence corresponding to the mature form of miRNA-224; *SLC4A4 3′-UTR*, a 20-mer RNA sequence for the 3′-UTR corresponding to SLC4A4 bearing the target site for miR-224; *SLC4A4 3′UTR-MM*, a negative control 3′UTR sequence for the SLC4A4 RNA, bearing 6 mismatches in the target site for miR-224, *anti-miR-224*, a modified antisense oligodeoxynucleotide complementary to the sequence of the mature form of miR-224; and *anti-miR-13MIS*, antisense oligodeoxynucleotides containing 13 mismatches, compared to anti-miR-224.

The sequence corresponding to the 3′-UTR of the gene was 20 nucleotides long to allow better visualization of the shifted band, to be more specific and to avoid secondary structures that longer sequences might acquire, and that most likely would interfere with the analysis.

All RNA and DNA oligonucleotides were purchased from Sigma-Aldrich (Madrid, Spain) and their specific sequences are listed in Table 1.

**Table 1:** Sequences of all the molecules used for the binding assays

**Molecule**	**Sequence**
**miR-224**	3′ UUGCCUUGGUGAUCACUGAAC 5′
**SLC4A4 3′-UTR**	5′ AAGCUUUCUAUUGUGACUUU 3′
**anti-miR-224**	5′ A*A*C*GGAAC*C*AC*T*AGT*GAC*T*T*G 3′
**anti-miR-13MIS**	5′ T*T*G*CCT AC*CAC*T*ACAC*T*G*A*A*G 3′
**SLC4A4 3′-UTR-MM**	5′ AAGCUUUCUAUU*CACUGAA*U 3′

Sequences of the RNA and DNA molecules used in this study. For antisense oligodeoxynucleotides, phosphorothioate-modified bonds were designed according to the minimal modification criteria [21] and are indicated as *. Mismatch bases are indicated in italics.

### Binding assays

Radiolabelled probes (20 000 cpm, [^32^P]) were incubated for 30 minutes at 37°C with the corresponding RNA or DNA molecules in the presence of 10 mM MgCl_2_, 100 mM NaCl, 50 mM HEPES pH 7.2 (AppliChem, Ecogen, Barcelona, Spain) and 5% Glycerol (Sigma-Aldrich, Madrid, Spain). Binding reactions were run in a 12% polyacrylamide gel (10 mM MgCl_2_, 5% Glycerol, 50 mM HEPES, pH 7.2) for 4 h at 190 V (4°C). Samples for denaturing gels were heated at 95°C for 5 min in loading buffer containing 95% formamide before electrophoresis in 5% polyacrylamide gels (1xTBE plus 8 M urea).

Gels were dried for 90 minutes at 80°C, exposed overnight to Europium plates and scanned in a Storm 840 scanner (Molecular Dynamics, GE Healthcare, Barcelona, Spain).

### MiRNA secondary structure prediction

The miR-224 secondary structures were predicted using the mfold program, v2.3, (http://mfold.rna.albany.edu/?q=mfold) [22]. This program determines the optimal and suboptimal secondary structures of RNA calculated for a 1 M NaCl solution at 37°C. The prediction was performed with the suboptimality parameter set at 5% so that it shows all the structures that have a free energy of formation of up to 5% higher than the optimal.

## Findings and discussion

**Figure 1:**
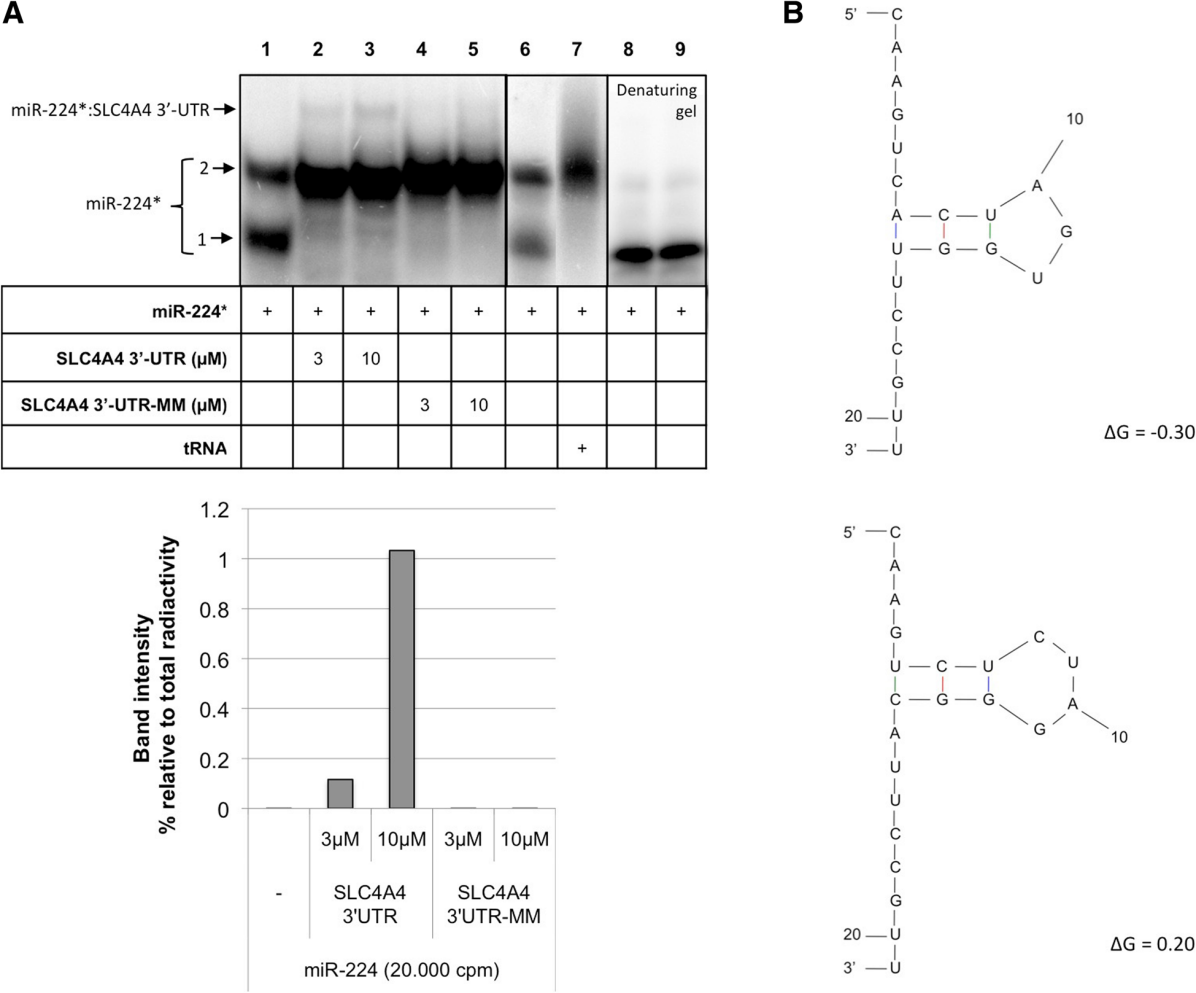
**Binding between miR-224 and the 3′-UTR of the gene *SLC4A4*. (A)** Lane 1 corresponds to the probe alone (miR-224*), lanes 2 and 3 correspond to the incubation of the probe with increasing concentrations of SLC4A4 3′-UTR (3 and 10 μM), lanes 4 and 5 correspond to the incubation of the probe with increasing concentrations (3 and 10 μM) of mutant SLC4A4 3′-UTR, lane 7 corresponds to the incubation of the probe with tRNA and lanes 8 and 9 correspond to the probe alone, for duplicate, on a denaturing gel. In the lower panel, the binding is measured by the increase in the intensity of the band corresponding to miR-224*:SLC4A4 3′-UTR and it is expressed as the percentage of intensity with respect to the total radioactivity in each lane after quantification with ImageQuant software. **(B)** Structure prediction of miR-224 conformations.

The first set of binding assays was performed using the radiolabeled miRNA-224 sequence (miR-224*) as the probe. MiR-224* displays two bands (Figure 1A, lane 1), one with higher mobility (band 1) and another band with lower mobility (band 2). When miR-224* was incubated with increasing concentrations of the 3′-UTR region of the gene *SLC4A4* (3 and 10 μM) we could observe the formation of a third band with lower electrophoretic mobility. This third band corresponds to the specific binding of the two molecules miR-224*:SLC4A4 3-UTR (Figure 1A, lanes 2 and 3) since it is not formed with SCL4A4 3′UTR-MM (lanes 4 and 5). Band number 1 switched to band number 2 in the presence of SLC4A4 3′-UTR, either the wild type or the mutant sequence. The conformation with faster electrophoretic mobility also disappeared in the presence of tRNA (lane 7). These results indicate that the upper band (lanes 2 and 3) represents the specific binding between miR-224 and SLC4A4 3′-UTR, rather than a multicomplex. MiR224 alone was also run on a denaturing gel to test whether or not the two bands present in native gels were two conformations of the same miRNA. As shown in Figure 1A (lanes 8 and 9), only one band was observed.

Thus, *in vitro*, miR-224 alone takes at least two conformations with different mobility. Structure prediction using the mfold software [22] showed different conformations with free energies of ΔG = -0.30 and ΔG = 0.20 (Figure 1B), indicating plasticity in the molecule. Therefore, the secondary structure of miRNA may alternate between conformations.

**Figure 2:**
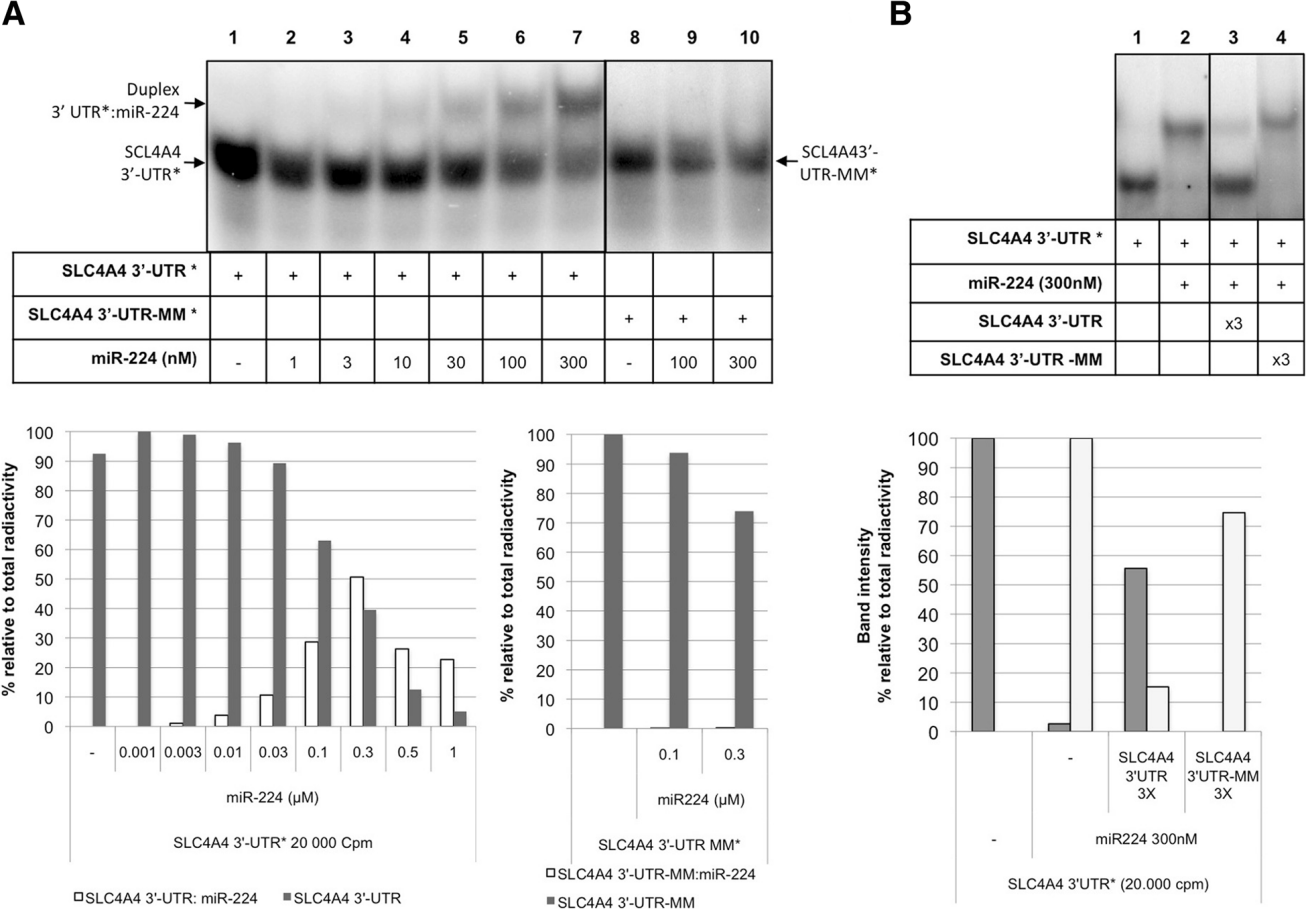
**Binding and competition between 3′-UTR of the gene SLC4A4 and miR-224. (A)** Lane 1 corresponds to the probe alone (SLC4A4 3′-UTR*) and the other lanes (2–7) correspond to the incubation of the probe with increasing concentrations of miR-224 (1nM-300nM). Lane 8 corresponds to the mismatch probe SLC4A4 3′UTR-MM* alone, lanes 9 and 10 correspond to its incubation with miR-224 (100 and 300nM). The intensity of the bands was quantified with ImageQuant software. Integrated in the panel below, it is represented the increase in the binding between SLC4A4 3′-UTR*:miR-224 (white bars) and the disappearance of the band corresponding to SLC4A4 alone (grey bars). Values correspond to the percentage of intensity of each band with respect to the total radioactivity in each lane. Probes and shifted bands are indicated by arrows. **(B)** The binding between SLC4A4 3′-UTR* and miR-224 was competed with unlabeled SLC4A4 3′-UTR and SLC4A4 3′-UTR-MM. SLC4A4 3′-UTR* was incubated with miR-224 either in the absence (lane 2) or the presence (lanes 3 and 4) of SLC4A4 3′-UTR and SLC4A4 3′-UTR-MM, respectively (3× the concentration of miR-224). The intensity of the bands was quantified with ImageQuant software. In the panel below, it is represented the corresponding decrease in the binding between SLC4A4 3′-UTR*:miR-224 (gray bars) and the increase of the band corresponding to SLC4A4 3′-UTR* alone (white bars). Values correspond to the percentage of intensity of each band relative to the total radioactivity in each lane.

The binding of miR-224 to its target gene was also confirmed using as radiolabeled probe the sequence corresponding to the 3′-UTR of *SLC4A4* (Figure 2A, lanes 1–7). When using SCL4A4 3′-UTR-MM sequence, the binding with miR-224 was not observed (Figure 2A, lanes 9 and 10). These results proved that miR-224 binds specifically to the 3′-UTR sequence of its target SLC4A4. Indeed, the experiments involving labeled 3′UTRs gave an easier read-out to understand (Figures 2A and 2B), where the bands appeared more defined and the proportion of binding was higher, which strongly supports a physical interaction of miR-224 with SCL4A 3′UTR *in vitro*.

To complete the usefulness of the assay we performed competition experiments of the interaction between miR-224 and SCL4A4 3′-UTR*. As shown in Figure 2B, this interaction was competed by wild type SCL4A4 3′-UTR but not by a mutated version of the sequence (mismatch).

When miR-224 was used as the probe, the necessary concentration of the corresponding unlabeled SLC4A4 3′-UTR sequence for the formation of the binding was 3 μM (Figure 1A, lane 2). However, when using SLC4A4 3′-UTR as the probe the necessary concentrations for the formation of such duplexes ranged from 10 to 300 nM of cold miR-224 (Figure 2A), therefore suggesting that the binding of miR-224 to a radiolabeled SLC4A4 3′-UTR is stronger and more specific than using miR-224 as probe.

**Figure 3:**
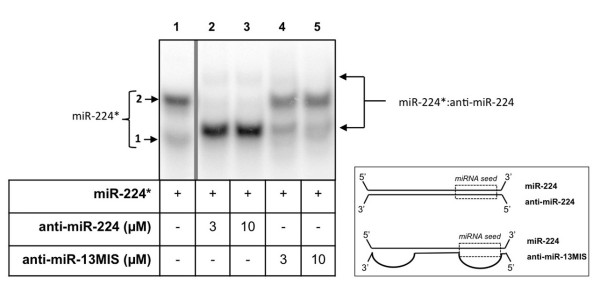
**Binding between miR-224 and anti-miR-224.** The probe miR-224* was incubated with anti-miR-224 (lanes 2–3), anti-miR-13MIS (lanes 4–5) at the specified concentrations. Probes and shifted bands are indicated by arrows. To the right it is represented a diagram showing the binding of the miR-224 to its anti-miR-224 by perfect matching, or to the negative control anti-miR-13MIS.

Next, miR-224 was incubated with anti-miR-224, the molecule used to successfully inhibit miR-224 function, and with the corresponding negative control used in functional assays, anti-miR-13MIS. This anti-miR contains 7 mismatches located in the miR-224 seed region and 6 additional mismatches in its 3′ region. As can be observed in Figure 3, miR-224 was bound by anti-miR-224 forming the corresponding miR-224*:anti-miR-224 duplex (lanes 2–3). A major band appeared between bands 1 and 2 of miR-224 alone (lane 1) and a more retarded band that might correspond to a different conformation of the duplex miR-224*:anti-miR-224. It is interesting to note that anti-miR-224 was also able to bind to the band number 2 of the probe alone corresponding to folded miR-224, at variance of SLC4A4 3′-UTR. This difference can be explained by the fact that anti-miR-224 is completely complementary to miR-224, in contrast to SLC4A4 3′-UTR, which has only 7nt complementary to the miRNA (miRNA seed). Therefore, anti-miR-224 can unfold miR-224* and form the observed duplex.

The effect of incubating miR-224 with anti-miR-13MIS can be observed in lanes 4 and 5 (Figure 3), showing that it is not able to bind miR-224, since it presents 13 mismatches with respect to miR-224, confirming its validity as a negative control.

**Figure 4:**
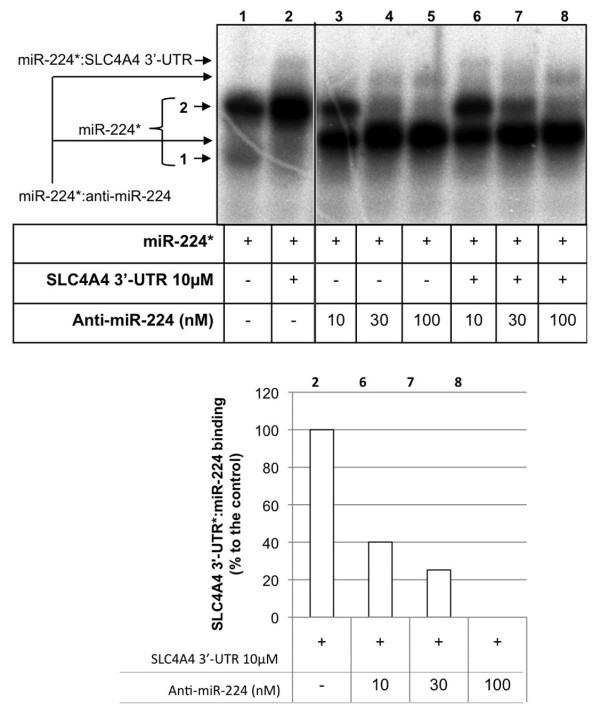
**Competition assay.** The binding between miR-224 and SLC4A4 3′-UTR was competed with increasing concentrations of unlabeled anti-miR-224. Probe miR-224* was incubated with SLC4A4 3′-UTR either in the presence (lanes 6–8) or the absence of anti-miR-224 (lane 2). Lanes 3–5 represent the binding between miR-224* and anti-miR-224. The intensity of the bands was quantified with ImageQuant software and normalized against the total radioactivity in each lane. It is represented the corresponding decrease in the binding between miR-224*:SLC4A4 3′-UTR, and it is expressed as a percentage of the control (lane 2) (lower panel).

Figure 4 shows the binding of miR-224* to SLC4A4 3′-UTR (lane 2) and the formation of the duplex miR-224*:anti-miR-224 after incubation with increasing concentrations of anti-miR-224 (lanes 3–5). In this case, the band pattern observed was the same as the one in Figure 3 (lanes 2 and 3). When competition experiments were performed in the presence of miR-224*, 3′-UTR SLC4A4 and anti-miR-224, it was observed that increasing concentrations of anti-miR-224 were able to displace the binding of miR-224* to the 3′-UTR of the gene *SLC4A4*. This was evidenced by the disappearance of the upper band corresponding to the binding of miR-224*:SLC4A4 3′-UTR (Figure 4, lanes 6–8 compared to lane 2). The competition between anti-miR-224 and SLC4A4 3′-UTR for the binding to miR-224* was also evidenced by the fact that higher concentrations of anti-miR-224 were needed to completely bind to miR-224 (lanes 6–7 compared to lanes 3–4).

## Conclusions

The results presented in this study show that EMSA is an easy, useful tool to validate in a direct and specific manner the interaction between a given miRNA and a determined cellular target mRNA, specially when using as probe the target 3′-UTR sequence rather than the miRNA. Using two synthetic RNA molecules corresponding to the mature form of a miRNA and to a fragment of the 3′-UTR region of a predicted target mRNA, this assay provides a view of the direct physical interaction between these two molecules.

## Abbreviations

3′-UTR: 3′-untranslated region; miR and miRNA: microRNA; SLC4A: Na/bicarbonate cotransporter 1; CDS2: CDP-diacylglycerol synthase (phosphatidatcytidylyltransferase) 2; HSPC159: Galectin-related protein; EMSA: Electrophoretic mobility shift assay.

## Competing interests

The authors declare that they have no competing interests.

## Authors’ contributions

AS, NM and XV performed the experimental work. VN and CJC helped with data interpretation and supervised the experimental work. All authors wrote and approved the final manuscript.
